# Robotic-Assisted unicompartmental knee arthroplasty restores native joint line height and reduces alignment outliers

**DOI:** 10.1007/s00264-025-06672-4

**Published:** 2025-10-15

**Authors:** George Mihai Avram, Horia Tomescu, Randa Elsheikh, Giacomo Pacchiarotti, Octav Russu, Vlad Rusu, Dennis Cicio, Andrej M. Nowakowski, Michael Tobias Hirschmann, Vlad Predescu

**Affiliations:** 1 Research Group Michael T. Hirschmann, Department of Clinical Research, Faculty of Medicine, University of Basel, Basel, Switzerland; 2 University Clinic for Orthopaedics & Traumatology, Kantonsspital Baselland Bruderholz, Bruderholz, Switzerland; 3 Faculty of General Medicine, Carol Davila University of Medicine and Pharmacy, Bucharest, Romania; 4 Department of Anatomy, Histology, Legal Medicine, and Orthopaedics, Sapienza University of Rome, Roma, Italy; 5 Orthopaedic and Traumatology Department, Pharmacy, Sciences and Technology George Emil Palade, University of Medicine, Targu Mures, Romania; 6 Faculty of General Medicine, University of Medicine, Pharmacy, Sciences and Technology “George Emil Palade” of Târgu Mureş, Targu Mures, Romania; 7 Faculty of General Medicine, University of Medicine, Pharmacy, Sciences and Technology “George Emil Palade” of Târgu Mureş, Targu Mures, Romania; 8 Faculty of General Medicine, “Iuliu Hațieganu” University of Medicine and Pharmacy, Cluj-Napoca, Romania; 9 University Clinic for Orthopaedics & Traumatology, Kantonsspital Baselland Bruderholz, Bruderholz, Switzerland; 10 Research Group Michael T. Hirschmann, Department of Clinical Research, Faculty of Medicine, University of Basel, Basel, Switzerland; 11 Orthopaedics and Traumatology Department, Ponderas Academic Hospital, Bucharest, Romania

**Keywords:** Robotic UKA, Conventional UKA, Joint line

## Abstract

**Purpose:**

Registry data suggests that robotic-assisted unicompartmental knee arthroplasty (rUKA) significantly reduces all-cause revisions compared to conventional implantation (cUKA). This study aims to compare joint line-related parameters and their reconstruction accuracy between rUKA and cUKA.

**Methods:**

Five databases were searched using a pre-defined strategy and inclusion criteria: (1) comparative studies reporting radiological outcomes, (2) human studies, (3) English language, and (4) meta-analyses for cross-referencing. Cadaveric or saw-bone studies were excluded. Data extracted included demographics data, pre- and postoperative radiological parameters (HKA, MPTA, LDFA, posterior tibial slope, femoral sagittal angle, joint line height, implant congruency), and outliers. A random-effects meta-analysis was conducted using mean difference (MD) and odds ratio (OR) as main effect estimators. Risk of bias was assessed using the Newcastle-Ottawa Scale (NOS), and publication bias was evaluated with funnel plots.

**Results:**

A total of 18 studies assessing 2470 patients (1112 rUKA, 1358 cUKA) were included in the analysis. No significant baseline differences were found in age, sex, BMI, follow-up period, MPTA, LDFA, or tibial slope. Postoperative radiological parameters showed no significant differences between groups for HKA, LDFA, MPTA, or tibial slope (*p* > 0.05). Joint line height was significantly lower in cUKA compared to rUKA (MD = -1.37 mm, 95% CI: -2.06 to -0.69, *p* < 0.001). Outlier analysis revealed that rUKA had significantly fewer outliers across relevant radiological parameters, including HKA, joint line height, tibial slope, femoral flexion, femoral implant congruency, and medial, anterior, and posterior tibial congruency.

**Conclusion:**

Reporting pre- and postoperative mean alignment parameters undermines patient-specific anatomy reconstruction with advanced technologies. Outlier reporting showed significant variability, with limited evidence supporting its clinical relevance. Future studies should focus on patient-specific reconstruction and define clinical thresholds for outliers.

**Supplementary Information:**

The online version contains supplementary material available at 10.1007/s00264-025-06672-4.

## Introduction

Unicompartmental knee arthroplasty (UKA) was introduced as a minimally invasive alternative to total knee replacement (TKA) for patients with end-stage osteoarthritis limited to one compartment of the knee [1]. Despite being associated with shorter operating times, better restoration of the knee kinematics, and improved functional recovery [2], high revision rates have been reported in UKA [3, 4]. The high revision rate has been primarily linked to aseptic loosening, which has in turn shown to be affected by component orientation and joint line restoration [5–7].

The advances in robotic-assisted surgery have provided the opportunity to improve bone preparation, component positioning, and ligamentous balance. Consequently, the use of robotic-assisted unicompartmental knee arthroplasty (rUKA) has been exponentially increasing. In the United States, up to 20% of UKA procedures are performed using robotic assistance and this number is projected to double in the next decade [8]. In addition to improving the precision of component position, rUKA has the potential to improve joint line restitution as compared to the conventional technique. Previous studies have shown that in conventional UKA (cUKA) the distalization of the joint line is inevitable, as it is lowered by implant thickness minus chondral thickness, with some studies reporting a joint line distalization of 4.3 mm in conventional technique and associating this to early implant failure [5].

To minimize the risk of loosening, resurfacing implants have been developed, in which the implant is placed directly on subchondral bone by minimizing femoral bone cuts [9]. Nonetheless, despite showing improved survival, the decreased bone resection carries the risk of changing joint line height, warranting greater tibial resection [10]. This, in turn, can shift the contact point of the femur to the periphery of the tibia, increasing the forces applied to the tibial surface, which predisposes to implant loosening and subsidence [7, 10]. While joint line restoration in conventional UKA is improved with standard implants and a true distal femoral cut [11], only two options for femoral cut width are allowed [10]. Conversely, rUKA allows for better customization of the femoral cut according to the position of the femoral component [6].

Although joint line restoration in rUKA has been investigated by numerous studies [6, 10, 12, 13], conflicting evidence exists, with some studies reporting no difference in joint line height after conventional and robotic UKA [11]. Based on this, this study aimed to assess the difference in joint line restitution in rUKA and cUKA through the conduction of a systematic review and meta-analysis. We hypothesized that rUKA is associated with less joint line distalization and an accurate tibial cut.

## Materials and methods

### Study selection and eligibility criteria

This systematic review and meta-analysis was conducted in accordance with PRISMA (Preferred Reporting Items for Systematic Reviews and Meta Analyses) guidelines and registered in PROSPERO under the registration n°CRD42024595082. Five databases were searched on the 24th of March 2024. The database search included (1) MEDLINE via PubMed, (2) Epistemonikos, (3) Cochrane Library, (4) Web of Science, and (5) Scopus.

The used search string strategy was as follows:

PubMed: Robot* AND (Unic* OR Partial OR UKA) AND Knee;

Web of Science: ((ALL = (Robot*)) AND (ALL = (Unic*) OR ALL = (UKA) OR ALL=(Partial))) AND ALL = (Knee));

Scopus: ALL (knee) AND ALL (robot*) AND ALL (unic*) AND (LIMIT-TO (LANGUAGE, “English”)) AND (LIMIT-TO (EXACTKEYWORD, “Human”) OR LIMIT-TO (EXACTKEYWORD, “Humans”) OR LIMIT-TO (EXACTKEY WORD,“Knee”) ORLIMIT-TO (EXACTKEYWORD,“Arthroplasty, Replacement, Knee”));

Epistemonikos: knee AND robot* AND uni* OR partial;

Cochrane: knee AND robot* AND uni* OR partial.

The inclusion criteria were as follows: (1) studies comparing rUKA and cUKA reporting radiological parameters or outliers (prospective or retrospective designs), (2) human studies, (3) meta-analyses for cross-referencing, and (4) English language. Studies reporting on navigation results, non-comparative studies, cadaveric studies, editorials, commentaries, surgical techniques, letters to the editor, or study protocols were excluded. Following the removal of duplicates, studies identified from databases were included in the selection process. Study selection was conducted by four independent reviewers and consisted of three stages: title screening, abstract screening, and full-text screening. For the title screening phase, the records were evenly divided, with two reviewers assigned to each half. Inter-rater agreement was calculated and reported as the intraclass correlation coefficient (ICC) with a 95% confidence interval. Discrepancies were resolved through discussion among all four reviewers.

During the cross-referencing phase, systematic reviews and meta-analyses identified were distributed among three of the reviewers, who extracted all studies meeting the inclusion and exclusion criteria. These were then compared against the studies identified in the initial literature search. No additional studies were identified during this step.

### Data extraction

The studies included for full-text screening were split in half and two reviewers were assigned for data collection for each half. The following information was extracted from eligible studies: authors, year, level of evidence, robot type, implant model, age, gender, BMI, follow-up, and operative time. Clinically relevant data points were as follows: (1) pre- and postoperative radiological parameters, and (2) radiological outliers. Only studies reporting on both preoperative and postoperative radiological parameters like hip-knee-ankle angle (HKA), medial proximal tibial angle (MPTA), lateral distal femoral angle (LDFA), and/or tibial slope (TS) were included in the final analysis. Similarly, outliers for HKA and joint-line height were reported only if the values of the parameters were also reported in the same studies.

### Statistical analysis and synthesis methods

The mean and standard deviation (SD), median, and range or 1st and 3rd quartile values were collected, when appropriate. The mean difference (MD) and 95% CIs were reported for continuous variables, while odds ratios (ORs) and 95% CIs were reported for categorical variables. A random-effects model was used for all forest plots due to the expected significant between-study heterogeneity and variability. The heterogeneity of the overall effect was assessed by computing the I^2^ estimate. The following thresholds for heterogeneity were used [14]: (1) 0–40%, heterogeneity is negligible; (2) 30–60%, moderate heterogeneity; (3) 50–90%, substantial heterogeneity; and (4) 75–100%, considerable heterogeneity.

When the sample mean and/or standard deviation were not reported, the transformation from median, range, and/or interquartile range was used as described by Wan et al. [15].

Publication bias was visually assessed using funnel plots which are presented in Supplementary Materials. The publication bias for each investigated forest plot is reported accordingly. The Newcastle-Ottawa scale was used to investigate the risk of bias [16]. Sensitivity analysis was not performed due to the limited number of identified studies. All statistical analyses were performed using SPSS v.30 (IBM SPSS Statistics for Windows, Version 26.0. Armonk, NY: IBM Corp). A *p* < 0.05 was considered statistically significant.

## Results

### Literature search

**Fig. 1:**
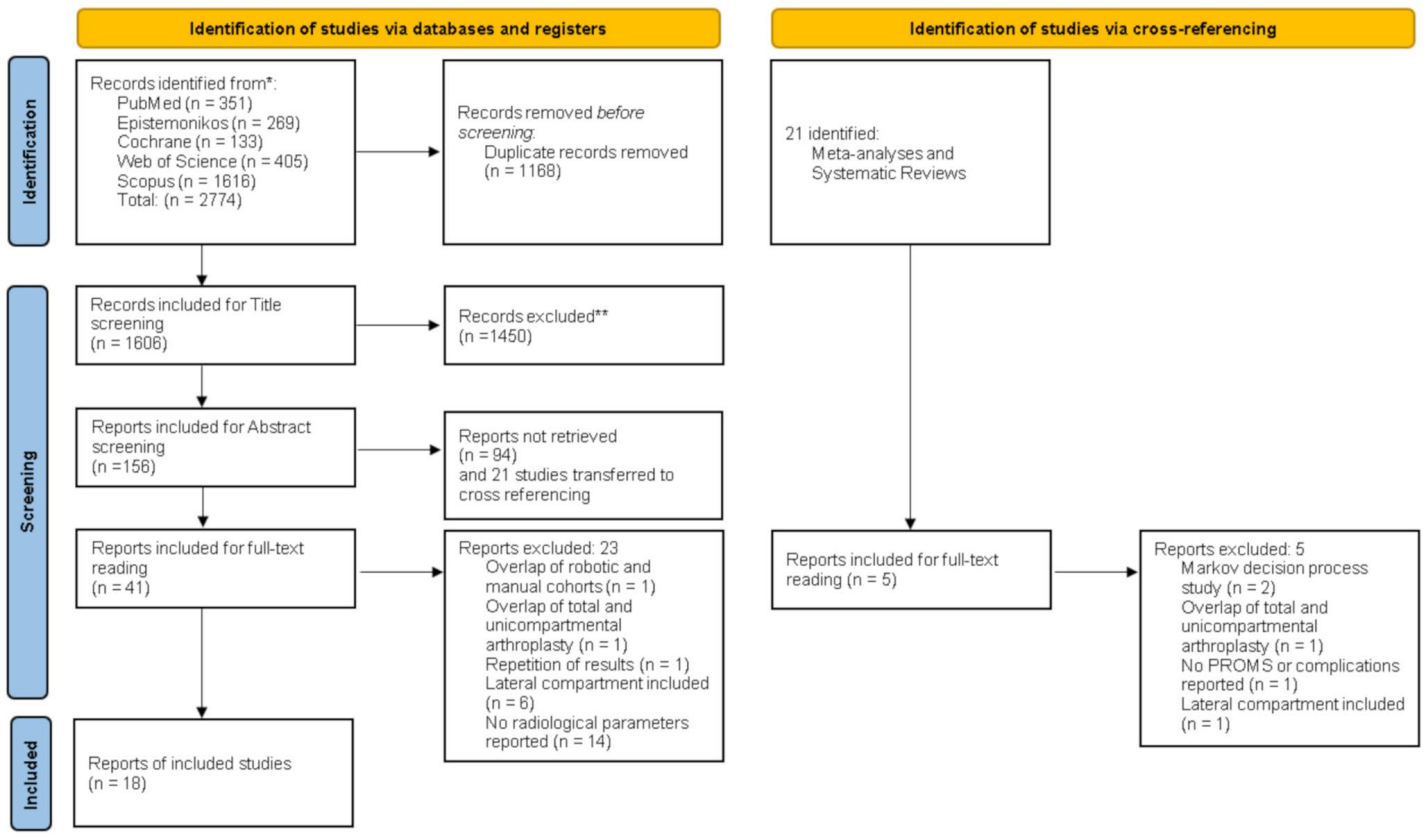
PRISMA flow diagram

A total of 2,774 studies were identified, and after removing duplicates, 1,606 articles were included for title screening. This was further narrowed down to 156 studies for abstract screening. The inter-rater agreement was 0.75 (95% CI: 0.73 to 0.77) for title screening and 0.83 (95% CI: 0.75 to 0.87) for abstract screening. 21 meta-analyses and systematic reviews were identified. No additional studies met the inclusion criteria from cross-referencing. Of the 41 studies included for full-text screening, 18 met the eligibility criteria and were included for final data extraction (Fig. 1). One of the studies included in the analysis compared a manual cohort with both Navio and MAKO separately and was referred to as “Batailler et al. (2021)a” and “Batailler et al. (2021)b” in our study [17].

### Study quality, bias, and characteristics

Risk of bias was assessed by one reviewer, using the Newcastle-Ottawa Scale (Table 1). seven out of 18 studies included in the analysis were classified as low quality, with NOS scores below 7. The remaining studies were deemed high quality, with NOS scores ranging from 7 to 9. Concerns regarding potential biases were identified, particularly in the selection of study participants, which may compromise the comparability of study cohorts. Additionally, there were notable risks of bias in outcome measurement, primarily due to inadequate follow-up protocols.

**Table 1:** Risk of bias assessment of included studies using the Newcastle-Ottawa scale

Study	Selection (4 max)	Comparability (2 max)	Outcome (3 max)	Total (9 max)
Thilak et al. (2020) [18]	★★★★	★	★	★★★★★★
Kwon et al. (2024) [19]	★★★★	★★	★★	★★★★★★★★
Park et al. (2019) [20]	★★★★	0	★★	★★★★★★
Yeung et al. (2023) [21]	★★★★	★	★★	★★★★★★★
Foissey et al. (2023) [22]	★★★★	★	★★★	★★★★★★★★
Cobb et al. (2006) [23]	★★★★	★★	★★	★★★★★★★★
Çabuk et al. (2022) [24]	★★★★	★	★	★★★★★★
Bell et al. (2016) [13]	★★★★	★★	★★	★★★★★★★★
Wu et al. (2021) [25]	★★★★	★	★★	★★★★★★★
Kazarian et al. (2021) [26]	★★★★	★	★	★★★★★★
Negrin et al. (2021) [27]	★★★★	★	★	★★★★★★
Hansen et al. (2014) [11]	★★★★	★	★★	★★★★★★★
Batailler et al. (2021) [17]	★★★★	★★	★★	★★★★★★★★
Batailler et al. (2023) [28]	★★★★	★★	★★	★★★★★★★★
Batailler et al. (2019) [12]	★★★★	★★	★★★	★★★★★★★★★
MacCallum et al. (2016) [29]	★★	0	★★★	★★★★★
Kumar et al. (2024) [30]	★★★★	★	★	★★★★★★
Herry et al. (2017) [10]	★★★★	★★	★★	★★★★★★★★

The majority of studies employed a retrospective comparative cohort design resulting in lower levels of evidence with only a few studies employing a prospective comparative design (Table 2).

**Table 2:** Studies included along with the respective study design and level of evidence.

Study	Study Type	Evidence Level
Thilak et al. (2020) [18]	Case Series	IV
Kwon et al. (2024) [19]	Retrospective cohort	III
Park et al. (2019) [20]	Retrospective cohort	III
Yeung et al. (2023) [21]	Case-Control	IV
Foissey et al. (2023) [22]	RCT	II
Cobb et al. (2006) [23]	RCT	II
Çabuk et al. (2022) [24]	RCT	II
Bell et al. (2016) [13]	RCT	II
Wu et al. (2021) [25]	Retrospective cohort	III
Kazarian et al. (2021) [26]	Retrospective cohort	III
Negrin et al. (2021) [27]	Retrospective cohort	III
Hansen et al. (2014) [11]	Retrospective cohort	III
Batailler et al. (2021)a [17]	Retrospective cohort	III
Batailler et al. (2021)b [17]	Retrospective cohort	III
Batailler et al. (2023) [28]	RCT	II
Batailler et al. (2019) [12]	Case-Control	IV
MacCallum et al. (2016) [29]	Prospective comparative	III
Kumar et al. (2024) [30]	Retrospective cohort	III
Herry et al. (2017) [10]	Case-Control	IV

Important variability was detected in terms of implant usage and patient sample size (Table 3).

**Table 3:** Implant model by study followed by the number of included patients

Study Name	Groups	Implant type	Patients number
Thilak et al. (2020) [18]	Manual	Oxford Biomet	12
	Robotic	Restoris	24
Kwon et al. (2024) [19]	Manual	Zimmer High Flex, Persona	35
	Robotic	Restoris	35
Wu et al. (2021) [25]	Manual	Zimmer High Flex	61
	Robotic	Restoris	52
Batailler et al. (2023) [28]	Manual	Journey 2 Uni	33
	Robotic	Journey 2 Uni	33
Foissey et al. (2023) [22]	Manual	HLS Uni Evolution	159
	Robotic	Journey	197
Park et al. (2019) [20]	Manual	Zimmer High Flex	57
	Robotic	Restoris	55
Yeung et al. (2023) [21]	Manual	Oxford Biomet	74
	Robotic	Restoris	74
Batailler et al. (2019) [12]	Manual	HLS Uni Evolution	57
	Robotic	HLS Uni Evolution	57
Cobb et al. (2006) [23]	Manual	Oxford Biomet	15
	Robotic	Oxford Biomet	13
Negrin et al. (2021) [27]	Manual	Journey	18
	Robotic	Journey	16
Herry et al. (2017) [10]	Manual	HLS Uni Evolution	23
	Robotic	HLS Uni Evolution	23
Kumar et al. (2024) [30]	Manual	Oxford Biomet	50
	Robotic	Journey	50
MacCallum et al. (2016) [29]	Manual	Zimmer High Flex, Miller-Galante, Journey	177
	Robotic	Restoris	87
Hansen et al. (2014) [11]	Manual	Zimmer High Flex	32
	Robotic	Restoris	30
Çabuk et al. (2022) [24]	Manual	Oxford Biomet	54
	Robotic	Restoris	36
Bell et al. (2016) [13]	Manual	Oxford Biomet	62
	Robotic	Restoris	58
Kazarian et al. (2021) [26]	Manual	Journey, Oxford Biomet	253
	Robotic	Restoris	86
Batailler et al. (2021)a [17]	Manual	HLS Uni Evolution	93
	Robotic	Restoris	93
Batailler et al. (2021)b [17]	Manual	HLS Uni Evolution	93
	Robotic	Journey 2 Uni	93

### Patient demographics

A total of 2470 patients were included from 18 studies, of which 1358 and 1112 were operated using manual and robotic-assisted techniques, respectively. There were no statistically significant differences in age, BMI, or follow-up period, although there was a significant difference in operative time (*p* = 0.01). Relevant patient demographics such as age, gender, operative time, and follow-up period are reported in Table 4.

**Table 4:** Demographics of both manual and robotic-assisted groups

	Manual	Robot	Mean Difference*	*p*
Total [10–13,17–30]	1358	1112	-	-
Age [11–13,17–20,22–28,30]	65.3 (8.75)	65.0 (8.5)	-0.08	0.87
Gender M/F (%) [11,17–20,22–25,27,28,30]	34/66	41.8/58.2	-	-
Operative Time [11,21–23,25,27,29]	81.0 (18.8)	86.5 (16.8)	12.68	0.01
BMI [11,12,17,19,20,22,26,28]	28.1 (4.4)	27.6 (3.8)	0.26	0.19
Follow-Up Period [11,12,19–23,25,27–29]	35.6 (20.3)	30.0 (10)	-7.62	0.23

*Calculated through inverse variance weighting

### Preoperative radiological measurements

Preoperative lower limb alignment as described by HKA, MPTA, LDFA, and TS showed statistical differences only in terms of HKA (*p* < 0.001) between the two groups as shown in Table 5.

**Table 5:** Preoperative HKA, MPTA, LDFA, and tibial slope

	Manual	Robot	Mean Difference	*p*
HKA [18–20,22,28]	175.1 (2.5)	174.1 (2.8)	-0.91	< 0.001
MPTA [25,28]	86.1 (2.6)	86 (2.4)	-0.08	0.83
LDFA [25,28]	86 (3)	86.3 (3.6)	0.13	0.73
Tibial slope [22,25]	80.8 (2.9)	81.1 (2.5)	0.33	0.61

### Postoperative radiological measurements and outliers

**Fig. 2:**
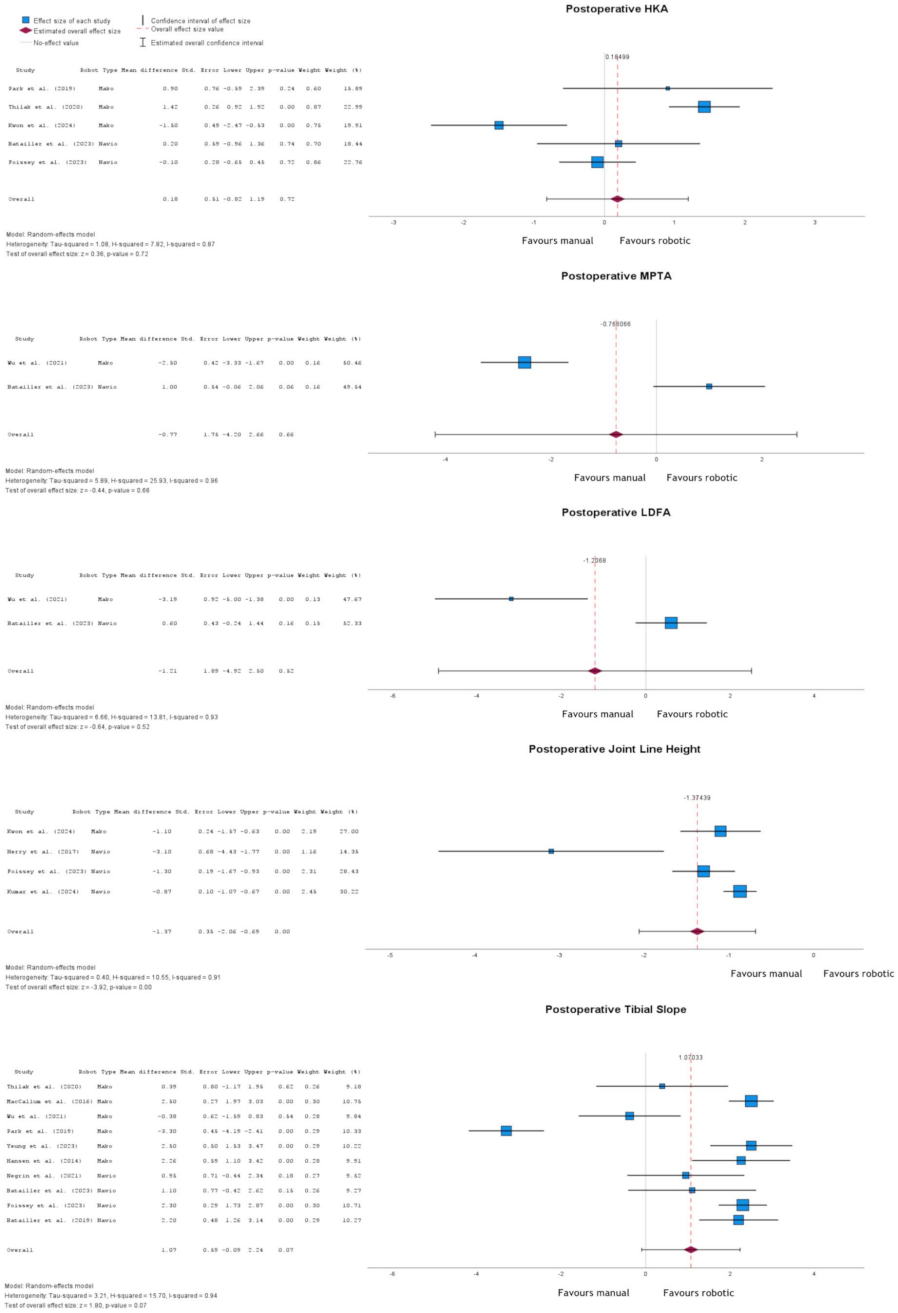
Forest plots of postoperative measurements: HKA, MPTA; LDFA, joint line height, and tibial slope

There was no statistically significant difference in postoperative HKA between the robotic and conventional groups (*p* = 0.72), mean difference of 0.18° (95% CI: -0.82 to 1.19) (Fig. 2). Postoperative MPTA had a mean difference of -0.77° (95% CI: -4.20 to 2.66; *p* = 0.66). LDFA also showed no significant difference (mean difference − 1.21°, (95% CI: -4.92 to 2.50; *p* = 0.52). Contrastingly, a statistically significant difference (*p* < 0.001) was detected for joint line height, with a mean difference of -1.37 mm (95% CI: -2.06 to 0.69). Postoperative TS resulted in a mean difference of 1.07° (95% CI: -0.09 to 2.24, *p* = 0.07).

**Fig. 3:**
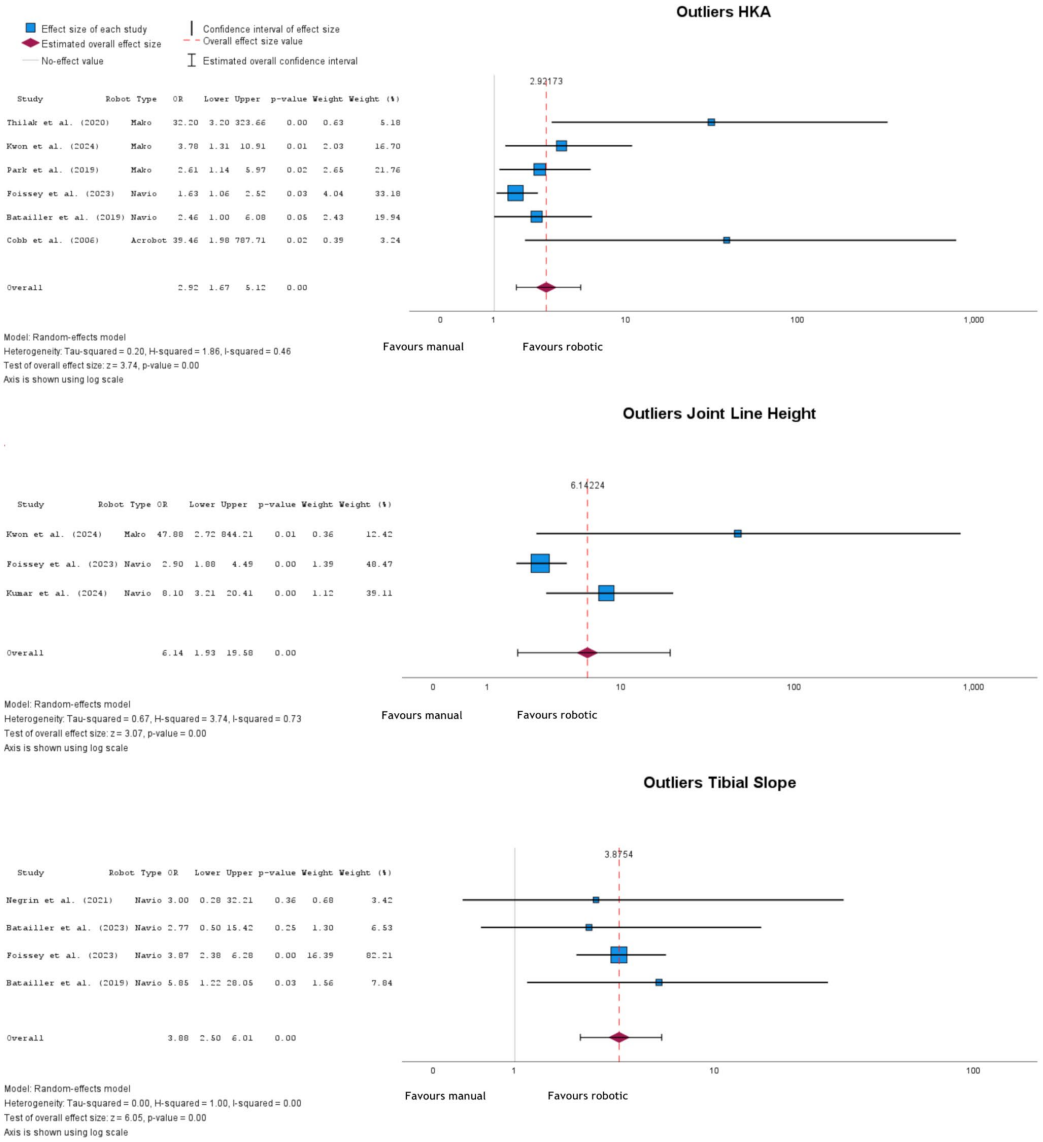
Forest plots of HKA outliers, joint line height outliers, and tibial slope outliers

A significant difference in outliers was found between cohorts for HKA, Joint Line Height, and TS, with manual implantation showing higher odds of outliers (Fig. 3). HKA outliers had an OR = 2.92 (95% CI: 1.67 to 5.12), *p* < 0.001. Joint Line Height outliers had an OR = 6.14 (95% CI: 1.93 to 19.50), *p* = 0.002, and TS outliers had an OR = 3.88 (95% CI: 2.50 to 6.01), *p* < 0.001.

**Fig. 4:**
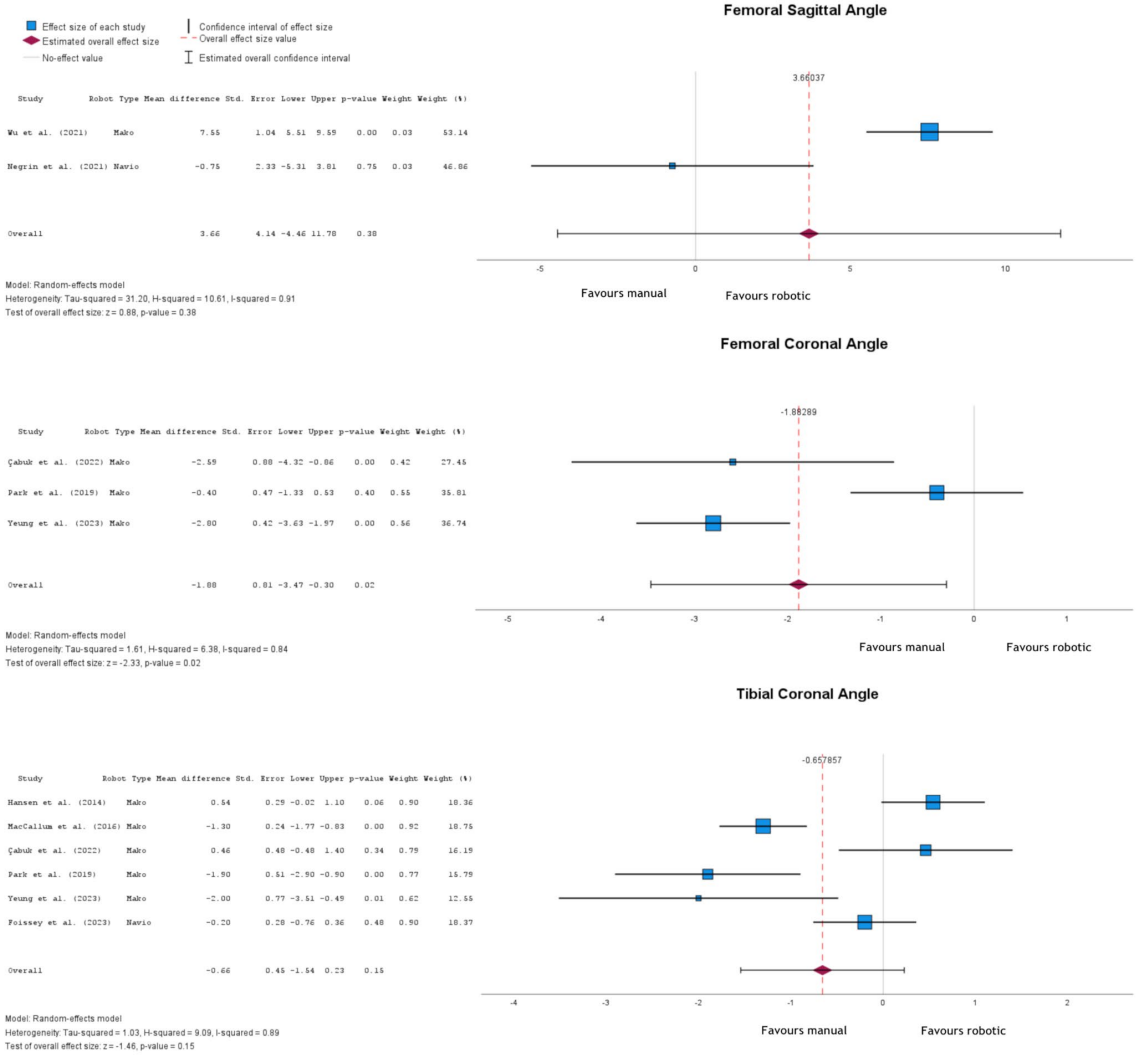
Forest plots of Femoral Sagittal, Femoral Coronal, and Tibial Coronal angles

There was no significant difference in postoperative femoral sagittal angle (*p* = 0.38) with a mean difference of 3.66° (95% CI: -4.46 to 11.70) in favour of the robotic-assisted group. On the other hand, when comparing the femoral coronal angle there was a statistically significant mean difference of -1.88° (95% CI: -3.47 to -0.30; *p* = 0.02), with a higher value after manual implantation. In terms of tibial component alignment, neither the tibial coronal angle nor the tibial sagittal angle was significantly different, with mean differences of -0.66° (95% CI: -1.54 to 0.23; *p* = 0.15) and 0.15° (95% CI: -1.19 to 1.49, *p* = 0.83) respectively (Fig. 4).

**Fig. 5:**
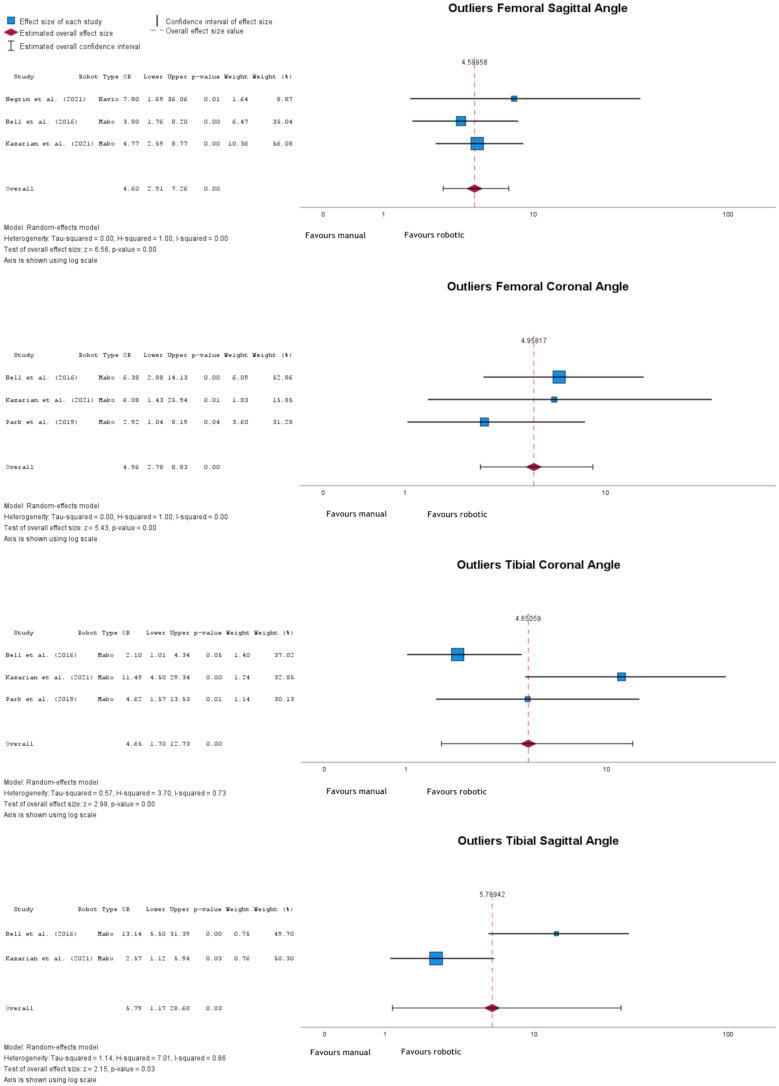
Forest plots of femoral coronal, femoral sagittal, tibial coronal, and tibial sagittal angles outliers

Outliers for femoral sagittal, femoral coronal, tibial coronal, and tibial sagittal angles were more frequent after manual implantation (Fig. 5). Femoral sagittal outliers had an OR = 4.60 (95% CI: 2.91 to 7.26), *p* < 0.001; femoral coronal outliers had an OR = 4.96 (95% CI: 2.78 to 8.83), *p* < 0.001; tibial coronal outliers had an OR = 4.65 (95% CI: 1.70 to 12.73), *p* = 0.003; and tibial sagittal outliers had an OR = 5.79 (95% CI: 1.17 to 28.60), *p* = 0.03.

**Fig. 6:**
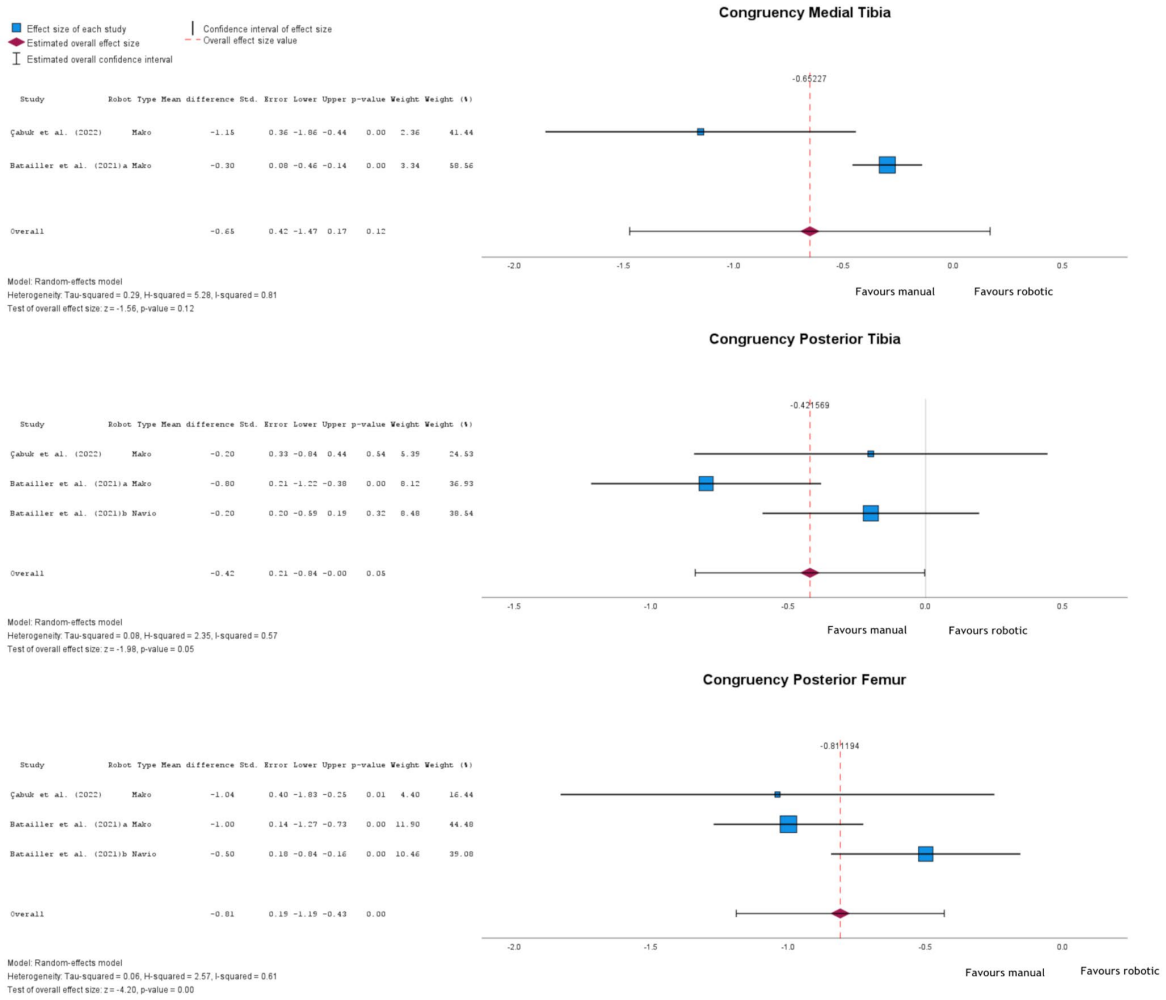
Forest plots of medial tibia, posterior tibia, and posterior femur congruencies

Robotic-assisted UKA consistently showed better congruencies than manual implantation, though not always statistically significant, with lower values for all but lateral tibia congruency, where both interventions yielded equal results (mean difference = 0, *p* = 1) (Fig. 6). Medial tibia congruency had a mean difference of -0.65 (95% CI: -1.47 to 0.17), *p* = 0.12. Posterior tibia congruency showed a significant difference (*p* = 0.05) with a mean difference of -0.42 (95% CI: -0.84 to 0.00). Posterior femur congruency showed a significant difference (*p* < 0.001) of -0.81 (95% CI: -1.19 to -0.43).

**Fig. 7:**
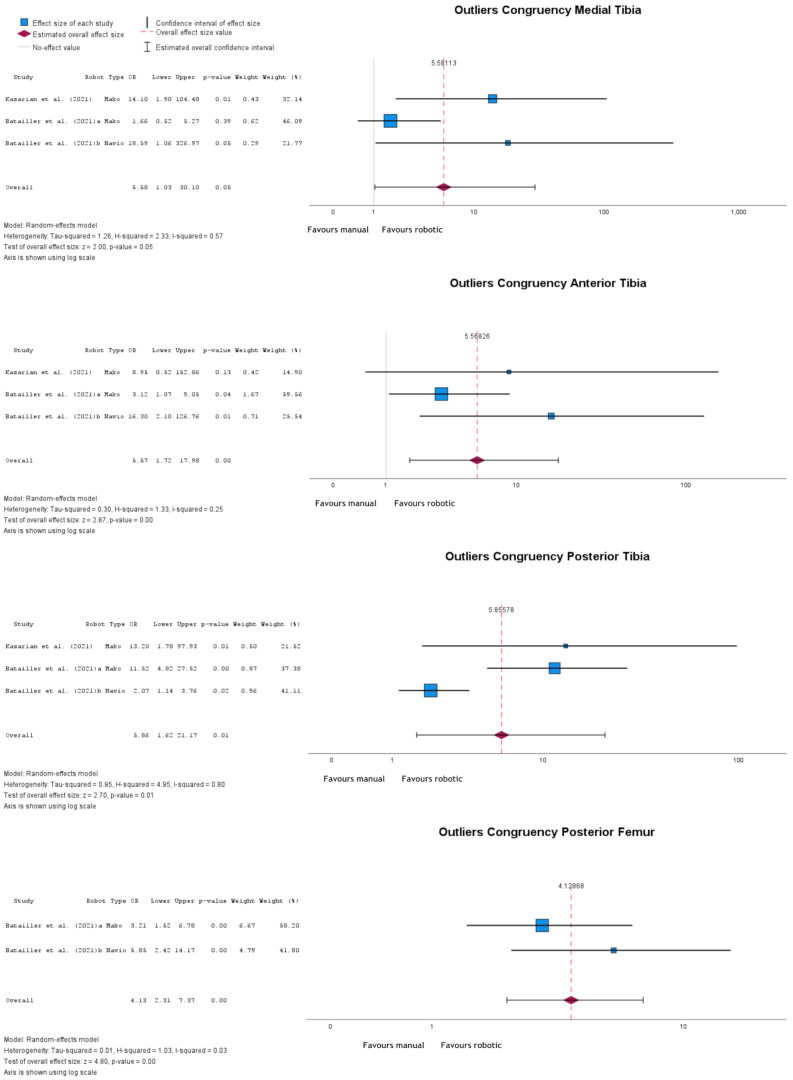
Forest plots of medial tibia, anterior tibia, posterior tibia, and posterior femur congruencies outliers

Out of the five reported congruency parameters, four had corresponding outliers: medial tibia, anterior tibia, posterior tibia, and posterior femur (Fig. 7). All four showed statistically significant odds ratios, with more outliers reported after manual implantation. Medial tibia congruency outliers had an OR = 5.58 (95% CI: 1.03 to 30.10), *p* = 0.05; anterior tibia outliers had an OR = 5.57 (95% CI: 1.72 to 17.98), *p* = 0.004; posterior tibia outliers had an OR = 5.86 (95% CI: 1.62 to 21.17), *p* = 0.01; and posterior femur outliers had an OR = 4.13 (95% CI: 2.31 to 7.37), *p* < 0.001.

## Discussion

The main finding of the present study is that conventional implantation is more often associated with implantation outliers in both the coronal and sagittal planes compared to robotic-assisted implantation. Although all investigated variables presented higher odds of outliers in the conventional implantation group, no significant mean postoperative differences were noted in terms of mean implantation values. This finding suggests that preoperative patient alignment parameters and implantation technique have a significant influence over postoperative alignment results. Importantly, outlier definition has proven to be highly inconsistent. In the analysis of HKA, two studies defined outliers as values more than 2° from the target [21, 30], one study used a threshold of more than 3° [12], and another study considered outliers as values falling outside the 175°-180° interval [25]. For joint-line height, all three studies reporting outliers used a threshold of more than 2° variation from the target values [21, 25, 29]. Similarly, for tibial slope, all four studies defined outliers as values outside the 82°-88° range [11, 13, 20, 25]. The thresholds for femoral sagittal angle outliers varied: one study used a 15° flexion threshold [24], another considered a 2° variation from target values based on the Biomet Operating Technique manual [23], and a third study defined outliers as values 3° away from a target of 45° [13]. Such an important heterogeneity suggests that measurement method as well as implant type significantly impact outlier definition, making comparisons across different arthroplasty systems very difficult. For femoral coronal angle, outliers were considered values outside of ± 3° from 90° [12], 10° from neutral [24], and 2° from the target values [24]. For tibial coronal angle, outliers were defined as values 2° from the target [23], 5° from neutral [24], and 3° from 90° [12]. Tibial sagittal angle outliers were considered values 2° from the target [23] or 5° from neutral [24]. Outliers for congruencies were reported by two studies: Kazarian et al. [26] and Batailler et al. [17] which consistently defined outliers as values outside of 2 mm for all congruency measurements, including anterior tibia, medial tibia, posterior tibia, and posterior femur. Kazarian et al. [26], on the other hand, defined outliers as follows: 2 mm for medial tibia and posterior tibia, and >3 mm for anterior tibia [24]. Despite their heterogeneous definitions, outlier incidence for each alignment variable is still a more sensible parameter than mean pre- and postoperative alignment values given that each patient’s transition from the preoperative to the postoperative state is much more important than the overall, sample mean value which diminishes the importance of patient-specific variations, making the two implantation methods difficult to compare.

Investigating joint line reconstruction in UKA is crucial, especially in light of recent nationwide reports showing a two-year revision rate of 4.8% (95% CI: 4.4–5.2), with patients under 55 having the highest revision rate at 6.3% (95% CI: 5.2–7.6) [31]. This trend is further confirmed by long-term data, which shows lower ten-year implant survivorship for patients under 55 years (82.6% to 85.6%) compared to older patient groups [32]. Given these findings, it is essential to consider the main revision reasons during preoperative planning and execution. Tibial loosening, a well-known failure mechanism often linked to a deep tibial cut and compensating with a thicker polyethylene insert, accounts for up to 27.9% of revised UKAs [31, 33]. The progression of osteoarthritis (OA) is the second leading cause of revision, responsible for 16.5% of cases. Additionally, femorotibial instability, which comprises 9.5% of revisions, is significantly influenced by bone cut thickness and alignment reconstruction [34]. Up-to-date evidence on patient-specific total knee arthroplasty implantation suggests that ligament balancing together with extension and flexion gap symmetries are closely related to performed bone cuts [35, 36]. Such data might also be applicable to UKA implantation in which limits in terms of posterior tibial slope and coronal alignment are still imposed [37]. Together, these factors highlight the importance of the “knee resurfacing” concepts in unicompartmental arthroplasty (UKA), which emphasize the need for precise implantation to optimize load distribution. While conventional implantation was not found to significantly lower mean accuracy, outlier data suggests that robotic-assisted implantation achieves better accuracy. The impact of implantation accuracy on revision rates is highly variable and dependent on the time frame [4], with some reports showing differences in revision rates between conventional and robotic UKAs, both with and without malpositioning [38]. This highlights that the success of UKA implantation is highly patient-dependant with pre- and postoperative mean values having only a secondary role.

The present study has identified several limitations in the literature that affect its generalizability. First, most of the studies primarily focused on reporting mean implantation values, with only a limited number of studies addressing outliers. This narrow focus on mean values may overlook the variability that exists within the data and limits the applicability of the findings to a broader patient population. Furthermore, the use of multiple robotic systems might hinder the ability to precisely identify error sources as robotic systems can employ different technologies to navigate the knee joint [39, 40]. Second, the definitions of outliers, as previously highlighted, varied significantly across studies. This inconsistency makes it difficult to draw reliable comparisons between studies, especially when considering the wide range of preoperative patient-specific characteristics and different implantation techniques that were used. Such variability further complicates efforts to generalize the results across diverse clinical settings. Third, the majority of the studies relied on AP (anteroposterior) and lateral X-rays to assess alignment parameters, with long-leg radiographs being used sparingly. This limited imaging approach may fail to capture the full extent of alignment issues, particularly when considering the broader context of joint mechanics [41]. As a result, femoral coronal angle and LDFA (lateral distal femoral angle) were reported separately, even though these angles are complementary and typically assessed together in comprehensive alignment evaluations (for example for functional knee phenotype and CPAK analyses). The lack of standardization in radiographic techniques may affect the accuracy and completeness of the data reported in these studies. Finally, the limited number of available comparative studies prevented the performance of a sensitivity analysis, which could have provided valuable insights into the robustness of the findings. This constraint also hindered a more thorough evaluation of publication bias, making it less noticeable in the overall analysis. The small number of studies available for review may have reduced the ability to detect significant publication bias, thus potentially influencing the study’s conclusions.

## Conclusion

Robotic-assisted UKA implantation results in significantly fewer alignment outliers and better joint line height restoration compared to conventional implantation. Additionally, robotic assistance optimizes implant sizing, leading to statistically significant improvements in implant-bone congruency. Future studies should focus on patient-specific anatomy reconstruction, provide more detailed information about the implantation techniques used, and establish a common framework for defining outliers.

## Supplementary Information

Below is the link to the electronic supplementary material.

Supplementary Material 1

## Data Availability

Data is provided within the manuscript or supplementary information files.
