# Supplementary material for: Robotic-Assisted unicompartmental knee arthroplasty restores native joint line height and reduces alignment outliers
Source: Int Orthop. 2025 Oct 15;49(11):2645–60. doi: 10.1007/s00264-025-06672-4 (PMC12594688; doi:10.1007/s00264-025-06672-4)
Supplement: Supplementary file 1 — Supplementary Material 1 [file 264_2025_6672_MOESM1_ESM.docx]

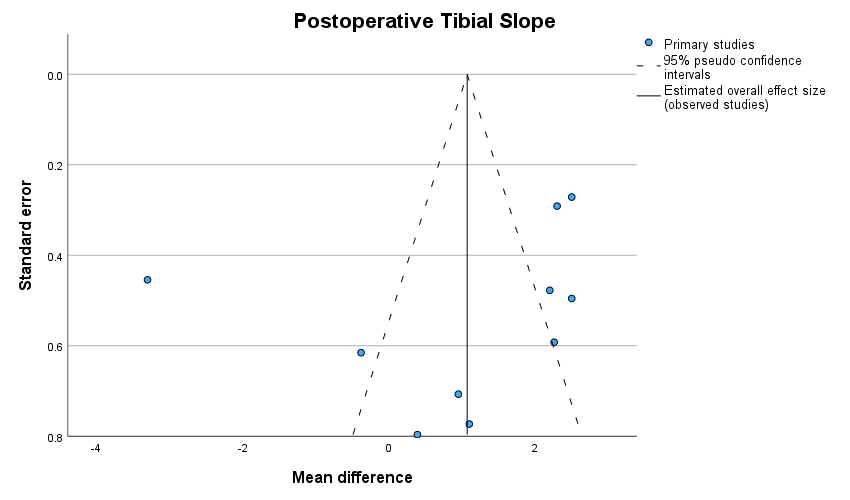

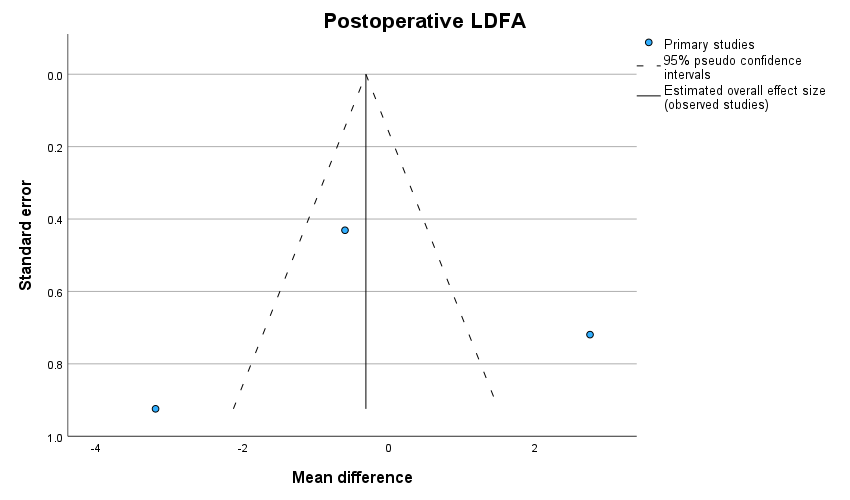

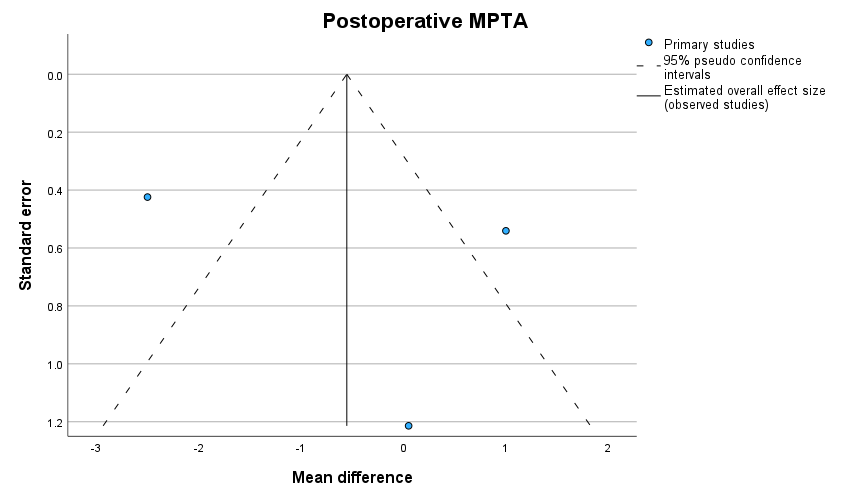

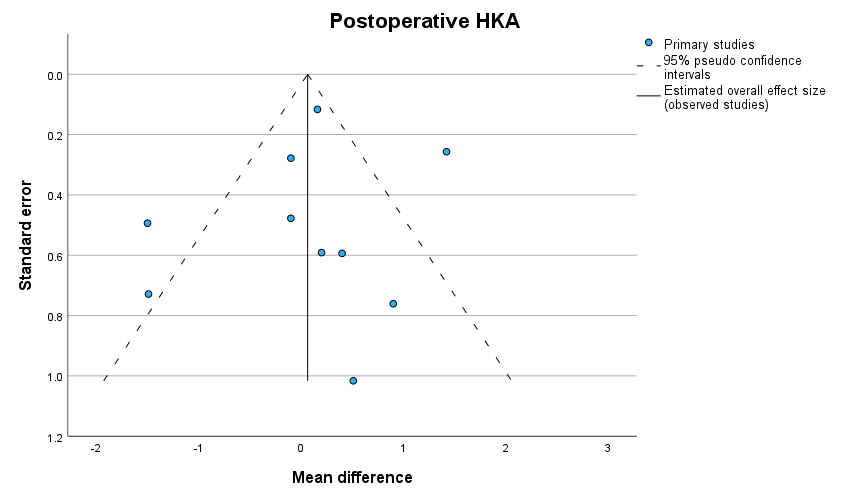
**Supplementary Figure 1.** Funnel plots of postoperative measurements (Figure 2) showing potential publication bias or small-study effects for HKA, MPTA, LDFA, and tibial slope. HKA: Hip Knee Ankle; MPTA: Medial Proximal Tibial Angle; LDFA: Lateral Distal Femoral Angle.


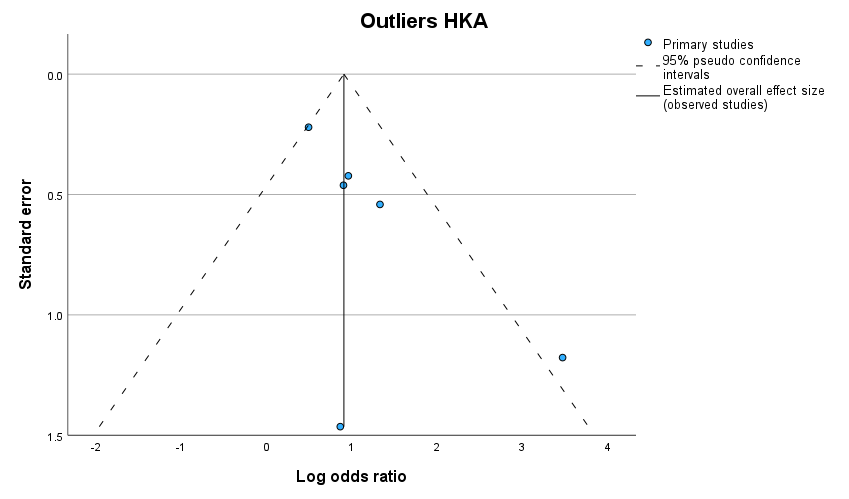

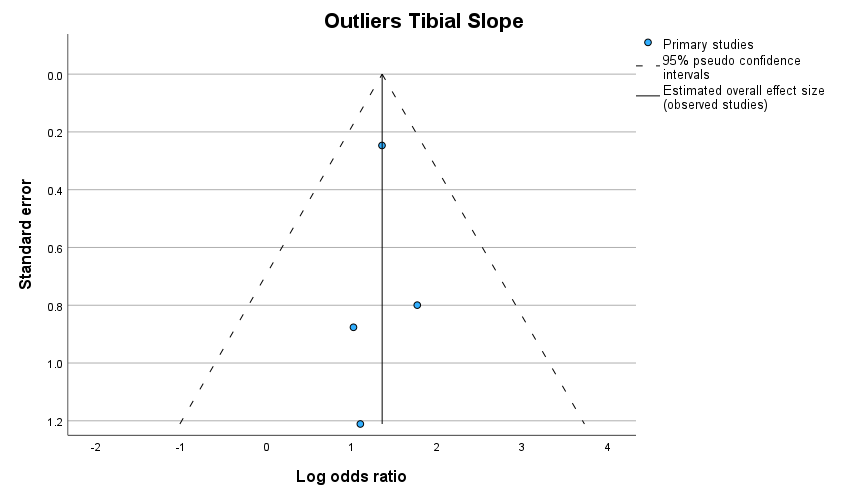

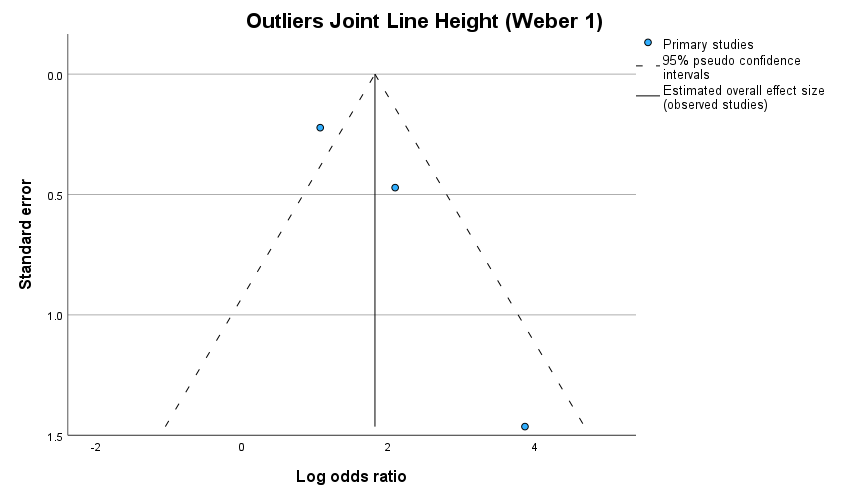


**Supplementary Figure 2.** Funnel plots of HKA, joint line height, and tibial slope outliers (Figure 3) showing potential publication bias or small-study effects for HKA and joint line height outliers. Low publication bias is observed for tibial slope outliers.


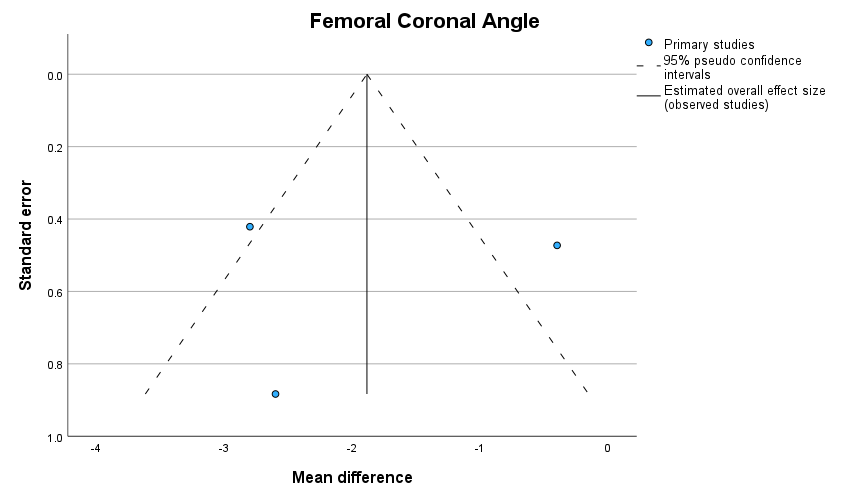

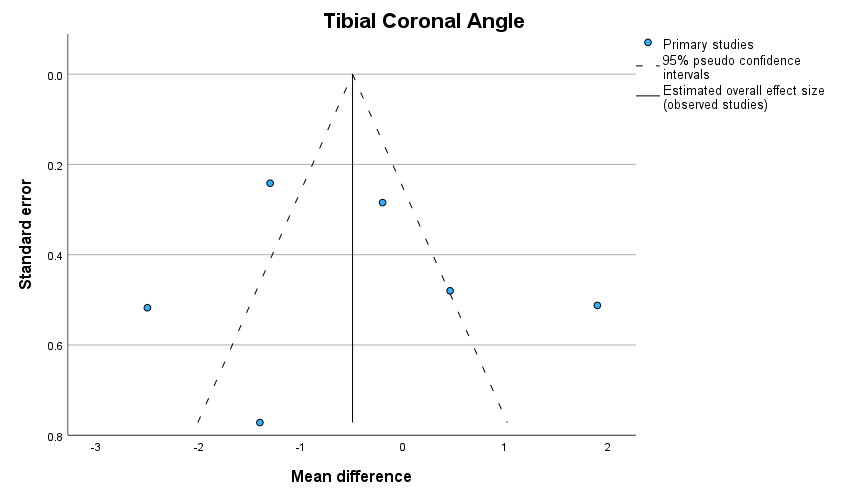

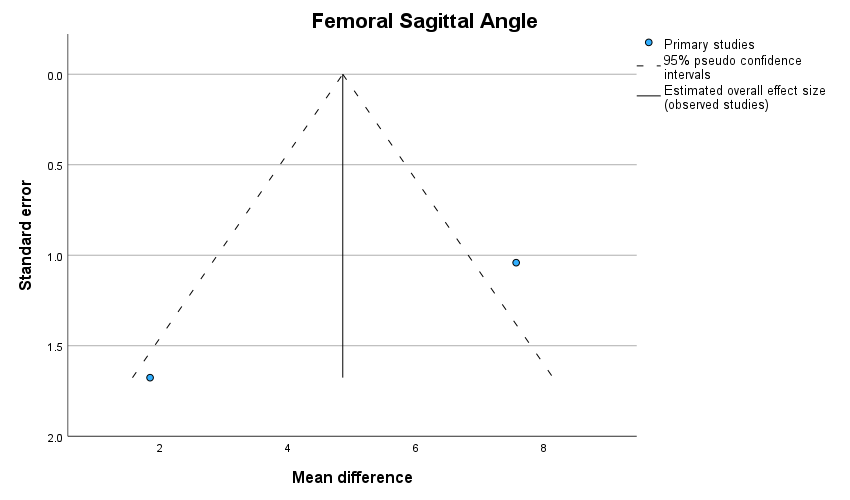


**Supplementary Figure 3.** Funnel plots of femoral sagittal, femoral coronal, and tibial coronal angles (Figure 4) showing potential publication bias or small-study effect for femoral coronal angle and even distribution of studies around the pooled effect estimate suggesting minimal publication bias or small-study effect for femoral sagittal angle and tibial coronal angle.


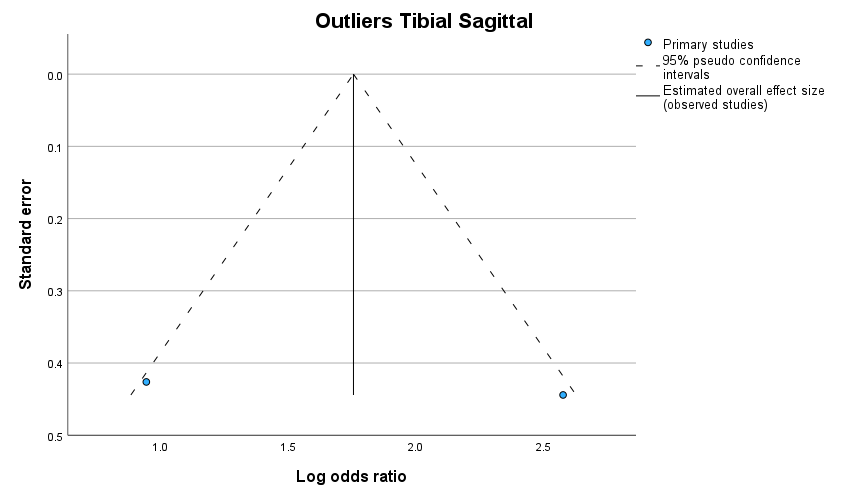

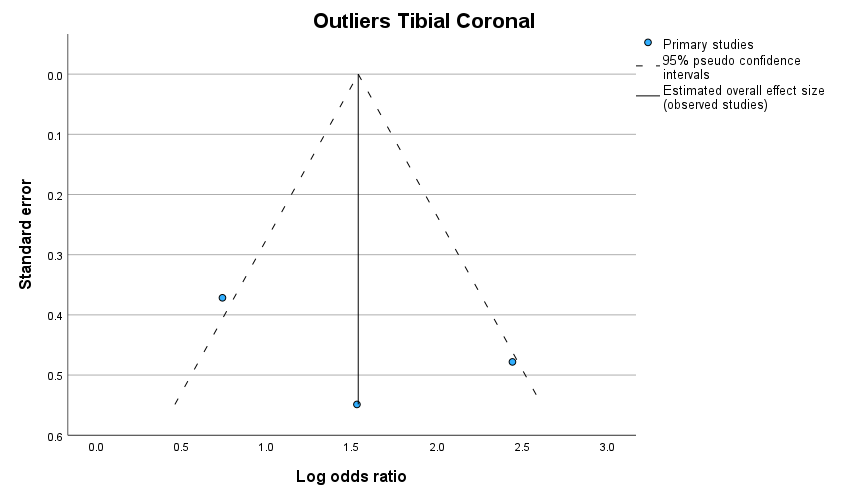

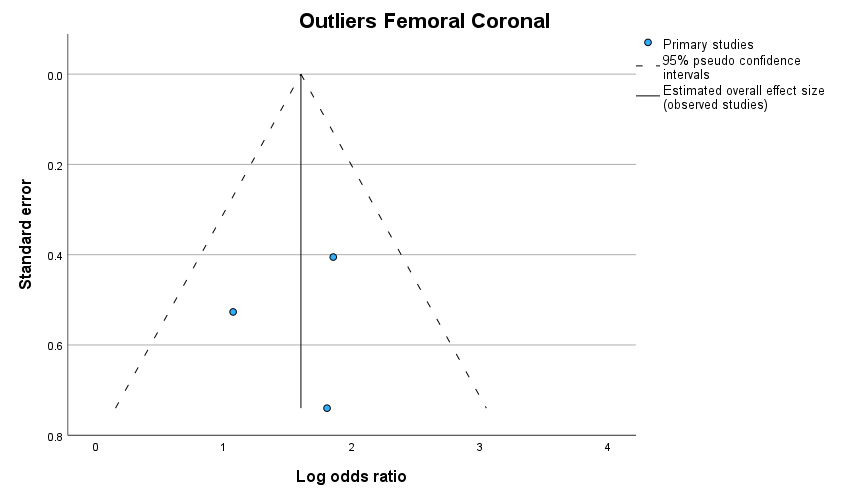

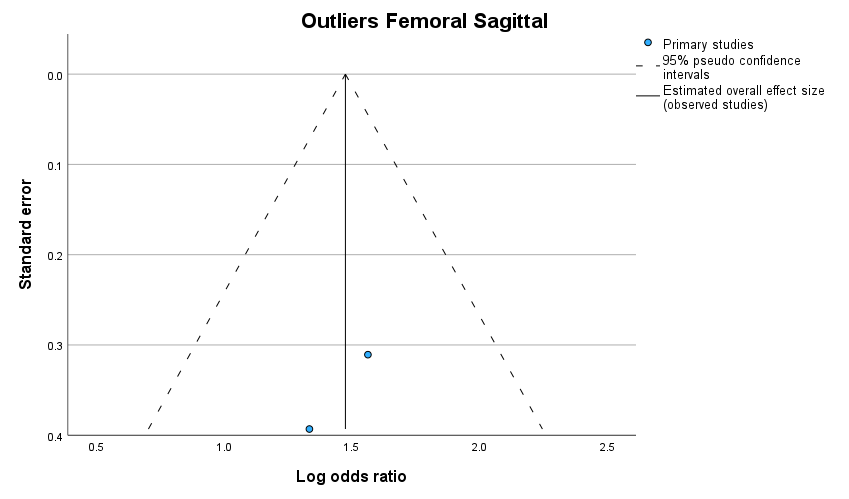
**Supplementary Figure 4.** Funnel plots of femoral coronal, femoral sagittal, tibial coronal, and tibial sagittal angles outliers (Figure 5) showing potential publication bias or small-study effect for femoral coronal angle outliers and even distribution of studies around the pooled effect estimate suggesting minimal publication bias or small-study effect for femoral sagittal, tibial coronal, and tibial sagittal angles outliers.

**
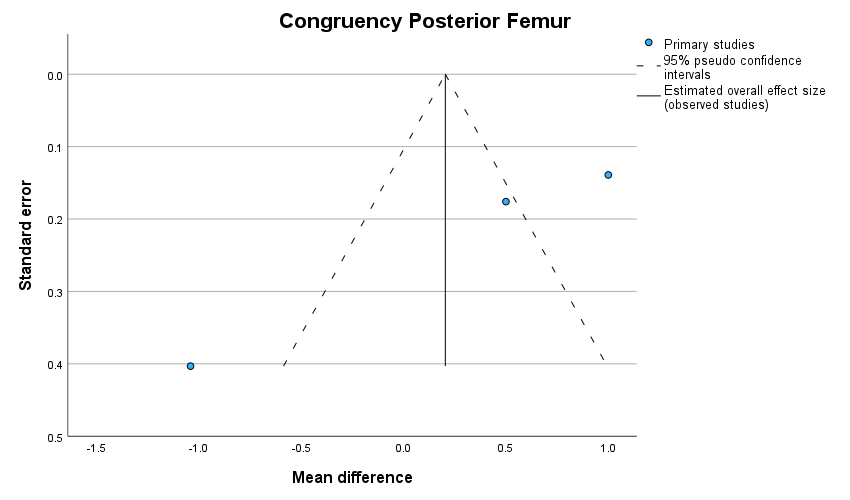
**
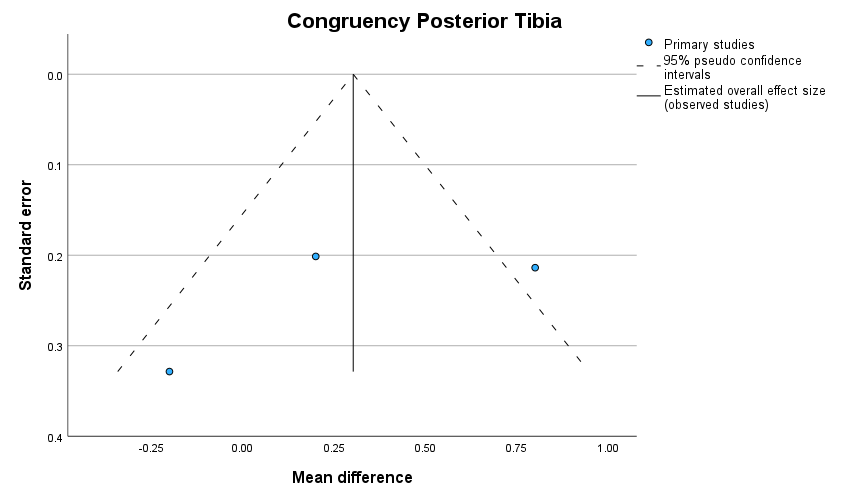

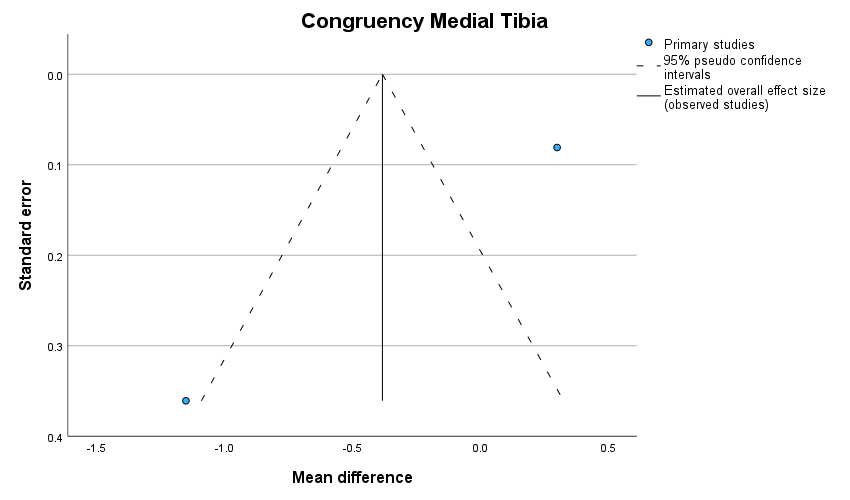


**Supplementary Figure 5.** Funnel plots of medial tibia, posterior tibia, and posterior femur congruency (Figure 6) showing potential publication bias or small-study effect for posterior tibia and posterior femur congruencies and even distribution of studies around the pooled effect estimate suggesting minimal publication bias or small-study effect for medial tibia congruency.


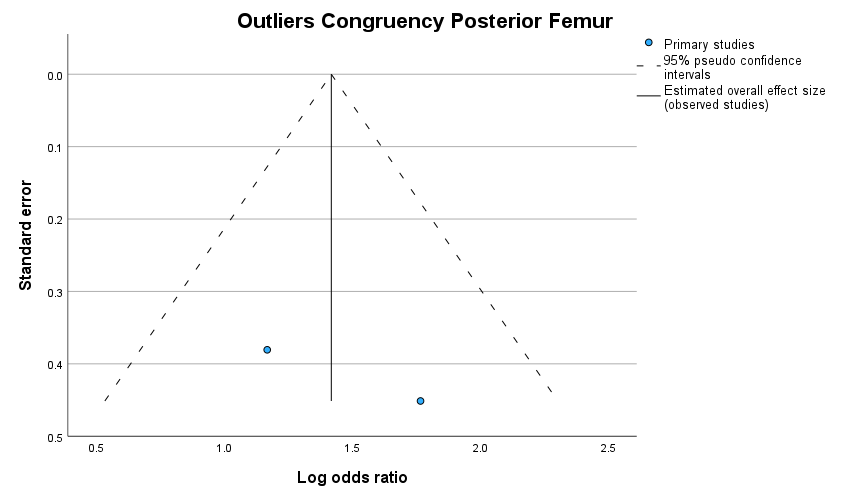

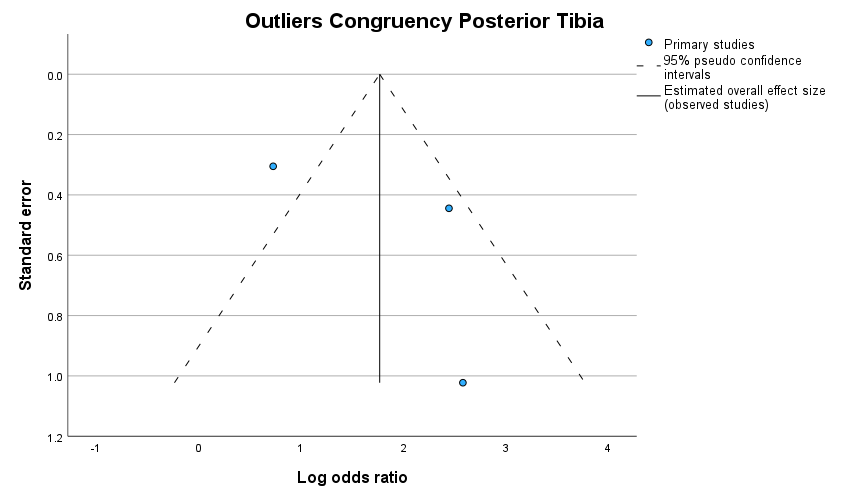

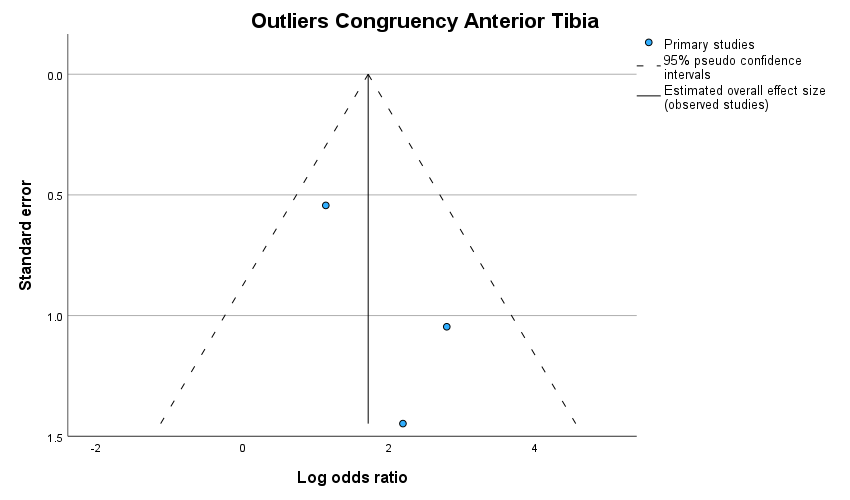

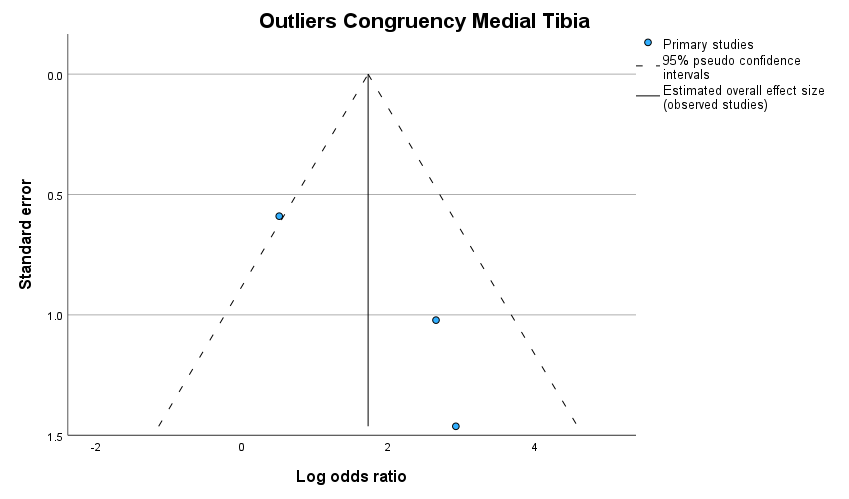


**Supplementary Figure 6.** Funnel plots of medial tibia, anterior tibia, posterior tibia, and posterior femur congruency outliers (Figure 7) showing potential publication bias or small-study effect for medial tibia, anterior tibia, and posterior tibia congruencies’ outliers and even distribution of studies around the pooled effect estimate suggesting minimal publication bias or small-study effect for posterior femur congruency outliers.
